# The Comparison of Anterior Segment Parameters in School-Aged Children with Laser-Treated Retinopathy of Prematurity, Untreated Preterm Birth, and Full-Term Birth

**DOI:** 10.3390/children13040546

**Published:** 2026-04-14

**Authors:** Barbaros Hayrettin Unlu, Canan Aslı Utine, Taylan Ozturk, Ceren Durmaz Engin, Aylin Yaman, A. Tulin Berk

**Affiliations:** 1 Department of Ophthalmology, Menemen State Hospital, 35660 Izmir, Turkey; barbarosunlu621@gmail.com; 2 Department of Ophthalmology, Dokuz Eylul University, 35340 Izmir, Turkey; cananutine@gmail.com; 3 Department of Ophthalmology, Izmir Tınaztepe University, 35400 Izmir, Turkey; ataylan6@yahoo.com; 4 Department of Ophthalmology, Izmir Democracy University, Buca Seyfi Demirsoy Education and Training Hospital, 35390 Izmir, Turkey; cerendurmaz@gmail.com; 5 Independent Researcher, 35210 Izmir, Turkey; aylinyaman@gmail.com

**Keywords:** autorefractokeratometer, cornea tomography, laser treatment, optical biometry, retinopathy of prematurity

## Abstract

**Background/Objectives**: To compare long-term refractive and anterior segment outcomes among preterm infants with retinopathy of prematurity (ROP) who underwent laser photocoagulation (LP), untreated preterm infants, and term-born controls. **Methods**: This study included 285 eyes of 144 patients aged 6–10 years, categorized into three groups: LP-treated type 1 ROP (Group 1), untreated preterms without type 1 ROP (Group 2), and full-term controls (Group 3). Data were collected, including birth weight (BW), gestational age (GA), ROP status, LP parameters (if required), as well as all ophthalmologic findings. Corneal tomography assessed keratometry, corneal thickness, corneal volume, anterior chamber metrics, and keratoconus indices. Optical biometry evaluated axial length (AL), keratometry, astigmatism (Ast), and anterior chamber depth (ACD). Cycloplegic autorefractometry measured spherical equivalent (SE), astigmatism (KAst), and keratometry. **Results**: Among 144 patients (63 female, 43.8%), the mean age was 8.7 ± 1.2 years, with a median GA of 34 weeks and BW of 2165 g. Significant differences were observed in corneal tomography parameters (Kh, Kv, Km, Kmax, astigmatism, and thickness metrics, *p* < 0.001), keratoconus indices (*p* < 0.001), optical biometry (AL, lens power, and ACD, *p* < 0.001), and cycloplegic autorefractometry (astigmatism and keratometry, *p* < 0.001) among the three groups. Refractive errors, including myopia and astigmatism, were most pronounced in laser-treated ROP cases (*p* < 0.036 and *p* < 0.001, respectively). **Conclusions**: Children with laser-treated ROP had steeper and thinner corneas, higher posterior astigmatism, and shallower ACD than preterm and term-born children. These findings likely reflect developmental anterior segment differences related to prematurity and treatment-requiring ROP, supporting careful long-term ophthalmologic follow-up.

## 1. Introduction

Retinopathy of prematurity (ROP) arises due to the abnormal proliferation of immature peripheral retinal vessels. Advances in neonatal care have improved the survival rates of extremely premature infants, leading to an increase in ocular problems, particularly ROP [1]. As the severity of ROP increases, so does the need for treatment, and current treatment options for ROP include cryotherapy, laser photocoagulation (LP), intravitreal anti-VEGF injections, and vitreoretinal surgery [2].

Preterm birth adversely affects ocular development, increasing the risk of refractive errors, strabismus, reduced contrast sensitivity, and visual field deficits. Refractive errors, including myopia and astigmatism, as well as altered corneal morphology, are more prevalent following preterm birth [3,4,5]. Previous studies have shown that not only the presence of ROP but also its treatment impacts ocular development [6]. In the long term, ROP may be associated with posterior segment conditions such as retinal holes, lattice degeneration, retinal detachment and retinoschisis [7,8]. Moreover, studies investigating the anterior segment parameters of preterm infants with and without ROP have identified variations in the cornea, anterior chamber, and lens dimensions [9]. However, only a limited number of studies have explored the effect of LP on corneal parameters in school-aged children [10]. This study analyzes a larger and older cohort than most existing studies, utilizing multiple devices to comprehensively measure corneal and anterior chamber parameters. Therefore, it aims to evaluate the impact of LP on anterior segment parameters by comparing preterm infants treated with LP, preterm infants who did not require treatment, and term-born infants aged between 6 and 10 years.

## 2. Materials and Methods

### 2.1. Study Population

This study included 285 eyes of 144 children aged 6–10 years who were examined in the Pediatric Ophthalmology Unit of our tertiary referral center between January 2020 and March 2024, and for whom parental consent for study participation had been obtained after the ethical committee approval. Their clinical records were retrospectively reviewed and analyzed in 2024 for the purposes of this study. Participants comprised children diagnosed with ROP, either treated or followed without treatment, preterm infants without ROP, and a control group of healthy, term-born children randomly selected from clinic examinations. Children with incomplete assessments due to poor cooperation, prior ocular surgeries other than LP, prior intravitreal anti-VEGF treatment, contact lens wear, or unrelated ophthalmologic pathologies were excluded. LP had been performed only in patients with type 1 ROP (laser-treated ROP group), and no other groups had a history of ocular laser treatment. None of the included children were undergoing amblyopia treatment, including atropine, penalization, or patching, at the time of examination. Both eyes were included when high-quality and reliable measurements were available in order to preserve sample size in this pediatric cohort. Analyses were performed at the eye level; therefore, potential within-subject inter-eye correlation should be considered when interpreting the results. However, in three children, all from the laser-treated ROP group, high-quality imaging could not be obtained in one eye; therefore, only the fellow eyes with reliable data were included in the analysis.

Neonatal ROP screening followed the Turkish Ophthalmology and Neonatology Societies’ guidelines, including infants born at ≤34 weeks GA or ≤1700 g BW, as well as those requiring cardiopulmonary support or deemed at risk by neonatologists [11]. Screening began at 4 weeks postnatally, but not before 31 weeks postmenstrual age. Patients were categorized into three groups: Group 1 (preterms with type 1 ROP who underwent LP), Group 2 (preterms not requiring LP), and Group 3 (term-born children). Data on BW, GA, systemic diseases, treatment timing, ROP zone and stage, plus disease, and LP parameters were retrospectively collected from medical records.

This study followed the Declaration of Helsinki, with approval from the Institutional Research Ethics Committee (decision no. 2021/19-54, 23 June 2021).

### 2.2. Evaluation of Anterior Segment Parameters

Best-corrected visual acuity (BCVA) was measured at 4 m using a Snellen chart and converted to logMAR units. Cycloplegic autorefractometry (Nidek ARK-530, Nidek Technologies, Gamagori, Japan) was performed 30 min after instilling 1% cyclopentolate eye drops in both eyes, and measurements of spherical, cylindrical, and axis values, horizontal keratometry (Kh), and vertical keratometry (Kv), as well as keratometric astigmatism (KAst) values were recorded. The spherical equivalent (SE) was calculated by adding half the cylindrical value to the spherical value. Refractive errors were defined as follows: myopia ≤ −1.00 D, myopia ≤ −3.00 D, hyperopia ≥ +1.00 D, hyperopia ≥ +3.00 D, astigmatism ≥ 1.00 D, and astigmatism ≥ 3.00 D.

Optical biometry (IOL Master 500, Carl Zeiss Meditec, Jena, Germany) was used to measure axial length (AL), anterior corneal keratometry values (K1, K2), astigmatism, anterior chamber depth (ACD), and SRK-T lens power.

Corneal tomography was performed with Pentacam (Oculus Optikgeräte GmbH, Wetzlar, Germany). The parameters measured included anterior corneal keratometry values (Kh, Kv, Km, Kmax), astigmatism and its axis, anterior and posterior corneal axes, thinnest corneal thickness (TCT), corneal vertex thickness (CVT), central corneal thickness (CCT), corneal volume (CV), anterior chamber volume (ACV), ACD, white-to-white (WTW) distance, and Pentacam-derived corneal shape indices, including ISV (index of surface variance), IVA (index of vertical asymmetry), KI (keratoconus index), CKI (central keratoconus index), IHA (index of height asymmetry), IHD (index of height decentration), Rmin (minimum radius of curvature), CSAI (corneal surface asymmetry index), and IS (inferior–superior index). The Belin–Ambrosio enhanced ectasia display/index, a composite Pentacam-derived parameter integrating anterior and posterior corneal elevation and pachymetric progression analysis for ectasia assessment, was not available retrospectively for all included patients and therefore was not included in the analysis.

### 2.3. Statistical Analysis

Statistical analyses were performed using SPSS Statistics version 28 (IBM, Armonk, NY, USA). Normality was assessed with the Kolmogorov–Smirnov test. Normally distributed data were reported as mean ± standard deviation, while non-normally distributed data were presented as median, minimum, maximum, and interquartile range. One-way ANOVA with post hoc Bonferroni tests was used for normally distributed continuous variables, while the Kruskal–Wallis H test with post hoc Dunn’s test was applied for non-normally distributed data. Categorical variables were analyzed using chi-square tests, with Pearson’s chi-square test applied when group frequencies exceeded five. A *p*-value < 0.05 was considered statistically significant.

## 3. Results

The study included 285 eyes of 144 participants with a mean age of 8.7 ± 1.2 years, comprising 63 females (43.8%) and 81 males (56.2%). The median GA was 34 weeks (range: 24–41 weeks), and BW was 2165 g (range: 530–4250 g). Group 1 consisted of 27 preterm infants treated with LP for ROP, while Group 2 included 59 preterms who did not require LP, of whom 44 had no ROP, 12 had bilateral ROP, and 3 had unilateral ROP. The control group (Group 3) included 58 randomly selected term-born children (≥37 weeks). GA and BW differed significantly among groups (*p* < 0.001), primarily due to differences in Group 3. Table 1 presents the demographic characteristics.

Cycloplegic autorefractometry revealed significant differences in Ast, Kh, Kv, and KAst. Ast was highest in Group 1 (−1.44 ± 1.14 D) compared to Group 2 (−0.61 ± 0.56 D, *p* < 0.001) and Group 3 (−0.77 ± 0.76 D, *p* < 0.001), with no difference between Groups 2 and 3 (*p* = 0.816). Kh was significantly greater in Group 1 (44.83 ± 1.61 D) than in Group 2 (43.57 ± 1.51 D, *p* < 0.001) and Group 3 (42.88 ± 1.73 D, *p* < 0.001), with Group 2 also exceeding Group 3 (*p* = 0.003). Kv followed the same pattern, being highest in Group 1 (46.37 ± 1.81 D), followed by Group 2 (44.58 ± 1.43 D, *p* < 0.001) and Group 3 (44.10 ± 1.75 D, *p* < 0.001), with Group 2 exceeding Group 3 (*p* = 0.028). KAst in Group 1 (−1.54 ± 0.83 D) was significantly more negative than in Group 2 (−0.97 ± 0.65 D, *p* < 0.001) and Group 3 (−1.22 ± 0.71 D, *p* < 0.001). Group 3 also showed more negative values than Group 2 (*p* = 0.025).

The comparison of BCVA among the groups showed significant differences (*p* = 0.021). Group 1 had poorer BCVA (0.033 ± 0.051) than Group 2 (0.014 ± 0.035, *p* = 0.017), while no significant differences were found between Groups 1 and 3 (*p* = 0.119) or between Groups 2 and 3 (*p* = 1.000). Group 1 had the highest prevalence of refractive errors, including myopia ≤ −1.00 D, myopia ≤ −3.00 D, astigmatism ≥ 1.00 D, and astigmatism ≥ 3.00 D, while no significant differences were found between Groups 2 and 3 (*p* = 0.036, *p* = 0.001, *p* < 0.001, and *p* = 0.001, respectively). Table 2 presents the refractive status comparisons.

Corneal tomography revealed significant differences in Kh, Kv, Km, and Kmax (*p* < 0.001). Kh was highest in Group 1 (44.40 ± 1.44 D) compared to Group 2 (*p* < 0.001) and Group 3 (*p* < 0.001). Anterior and posterior astigmatism also differed significantly (*p* < 0.001 and *p* = 0.01, respectively), with anterior astigmatism highest in Group 1 (1.62 ± 0.85 D). CCT, CVT, and TCT values were significantly different across groups (*p* < 0.001), with Group 1 having the thinnest CCT (545.8 ± 33.7 µm). No significant differences were found in CV (*p* = 0.081), but WTW, ACV, and ACD values differed significantly (*p* < 0.001). Table 3 details the corneal tomography comparisons.

Significant differences were found in ISV, IVA, KI, and CKI median values among the groups (*p* < 0.001, *p* = 0.008, *p* < 0.001, and *p* = 0.001, respectively). ISV was highest in Group 1 (24.54 ± 6.72). Rmin was significantly lower in Group 1 (7.26 ± 0.30) compared to Group 2 (*p* < 0.001) and Group 3 (*p* < 0.001). IVA was also highest in Group 1, while IHA showed no significant differences (*p* = 0.399). Significant differences were observed in IHD, Rmin, CSAI, and IS (*p* = 0.024, *p* < 0.001, *p* = 0.043, and *p* < 0.001, respectively). Table 4 presents the Pentacam KCN indices comparison.

The median AL values differed significantly among the groups (*p* < 0.001), with Group 1 having the shortest AL (21.9 (20.3–25.3) mm) compared to Group 2 (22.8 (21.4–25.3) mm, *p* < 0.001) and Group 3 (22.9 (20.7–25.1) mm, *p* < 0.001). Significant differences were also found in SRK-T lens power, with Group 1 showing the highest value (24.24 ± 3.57 D). ACD was shallowest in Group 1 (3.19 ± 0.37 mm) compared to Group 2 (*p* < 0.001) and Group 3 (*p* < 0.001). Table 5 provides a comparison of IOL Master measurements.

## 4. Discussion

This study observed significant anterior segment changes by school age among children who underwent LP for ROP treatment, compared to preterm infants without any need for ROP treatment and healthy term infants.

Several studies indicate that both prematurity and ROP affect long-term refractive and optical functions, alter corneal curvature and anterior segment parameters, and may lead to amblyopia [6,12]. Zhu et al. [3] reported steeper corneal curvature in untreated mild ROP cases at age 6, while Wu et al. [13] found significantly steeper corneas in both untreated preterms and those with ROP compared to term-born controls, emphasizing prematurity’s stronger effect over laser therapy. Chen et al. [14] demonstrated that preterm birth contributes to refractive errors associated with shorter ACD, greater lens thickness, and steeper corneas. Similarly, the German Gutenberg Health Study linked low birth weight to a steeper and thinner cornea with a smaller diameter [5]. However, we observed steeper K values in the non-laser-treated preterm group than in the term-born control group and even steeper values in the laser-treated ROP group than in the non-laser-treated preterm group, suggesting that corneal steepening was most pronounced in the laser-treated ROP group. However, this finding cannot be interpreted as an independent effect of laser treatment, given the greater severity of prematurity and ROP in this group. This discrepancy may arise from differences in age at assessment, measurement techniques, ROP severity, treatment protocols, and genetic or environmental factors influencing ocular development.

Studies have reported higher corneal astigmatism in preterm infants, especially in untreated ROP cases, with further increases in laser-treated cases over time. Wu et al. [13] found that preterm birth and LP therapy increased anterior astigmatism, while posterior astigmatism was higher in untreated preterms, with no significant effect from LP. Keratoconus is characterized by corneal thinning and steepening, making early diagnosis crucial [15]. Aslani et al. [16] demonstrated that anterior astigmatism is more relevant in detecting KCN severity, while posterior astigmatism is more affected in early disease stages, suggesting it as a potential biomarker for corneal steepening. In our study, anterior astigmatism was significantly higher in the laser-treated group than in the untreated preterm group and term-born controls (*p* < 0.001 and *p* = 0.007, respectively), with no difference between the non-laser-treated preterm group and the term-born control group. Posterior astigmatism was higher in the laser-treated ROP group than in the non-laser-treated preterm group (*p* = 0.023) but showed no significant difference between the non-laser-treated preterm group and the term-born control group (*p* = 0.05). Given that posterior astigmatism has been proposed as a potential early marker of corneal shape alterations, its higher levels in laser-treated eyes may reflect differences in corneal development.

Recent studies suggest that laser therapy may influence the natural corneal flattening process during emmetropization, potentially leading to steeper corneal profiles. Fielder et al. [17] proposed that preterm infants have a “thermal defect” impairing postnatal ocular growth, contributing to reduced corneal flattening and increased myopia risk. Animal studies further support the role of altered thermal gradients in myopia development [18]. These findings suggest that both prematurity-related factors and treatment exposure may be associated with variations in corneal development and geometry. However, their independent contributions cannot be distinguished in our study design.

Genç et al. [19] reported myopia (≤−1.00 D) in 29.1% of preterm infants and 55% of laser-treated ROP cases, while 23.5% had myopia of ≤−4.00 D. Another study found higher myopia rates in untreated ROP cases and preterm infants without ROP compared to term-born controls at 7 years [8]. Zhu et al. [3] also observed more frequent myopia in untreated preterms. However, our study found no significant differences in the prevalence of myopia (≤−1.00 D) and higher degrees of myopia (≤−3.00 D) between untreated preterm and term-born groups. However, this finding should be interpreted with caution, as the untreated preterm group included both children without ROP and those with spontaneously regressed, untreated ROP, which may have obscured subgroup-specific differences. Nevertheless, both untreated and laser-treated ROP have been associated with a higher prevalence of myopia, which may be related to alterations in normal ocular development, impaired emmetropization, and changes in anterior segment configuration rather than axial elongation alone. Mechanisms such as scarring, inflammation, and scleral remodeling following laser treatment may further contribute. Compared to anti-VEGF therapy, laser treatment is more strongly linked to higher myopia severity, which may explain our findings [20].

Kirwan et al. [21] observed greater corneal thickness in infants born at 31 weeks compared to term-born infants, with a subsequent decrease by term. Long-term findings; however, vary. Fieß et al. [9] found no significant long-term differences in corneal thickness after prematurity or ROP, while Wu et al. [13] reported lower TCT in preterm groups compared to term-born controls, regardless of ROP, and noted thinner corneas in untreated preterm cases than in laser-treated cases. In our study, CCT, CVT, and TCT were significantly thinner in the laser-treated ROP group than in the non-laser-treated preterm group and the term-born control group (*p* < 0.001). These findings align with keratometric values and astigmatism differences, showing that corneal thinning was most pronounced in the laser-treated ROP group; however, this finding cannot be attributed solely to laser treatment, as this group had more severe prematurity and ROP. This may be associated with corneal shape changes accompanying steepening, which may also result in reduced central thickness, or inflammatory and tissue remodeling processes that may occur following photocoagulation [10].

Pentacam-derived corneal shape indices are commonly used in the tomographic assessment of keratoconus; however, their interpretation in school-aged children born prematurely, especially those with a history of treatment-requiring ROP, remains uncertain [22]. In our study, several of these indices differed significantly among groups, with the laser-treated ROP group showing steeper anterior curvature, thinner corneas, and altered corneal shape parameters. Fieß et al. [9] also found higher IVA, IHD, and Rmin values in preterm infants with ROP but no differences in the Belin–Ambrosio Index. However, these findings should not be interpreted as evidence of keratoconus or a definite ectatic tendency. Rather, they may reflect a developmental anterior segment phenotype related to prematurity, abnormal ocular growth, and the severity of ROP/treatment history. Because pediatric normative values for these indices are limited, particularly in former preterm children, keratoconus-based diagnostic thresholds are not directly applicable in this setting. Therefore, these tomographic findings should be considered descriptive of corneal morphology rather than indicative of a distinct corneal ectatic disorder.

Optical biometry and corneal tomography in our study showed significantly shorter AL, higher SRK-T lens power, shallower ACV and ACD, and smaller WTW in the laser-treated ROP group compared to other groups (*p* < 0.001 for all). While some studies reported longer AL and lower SRK-T lens power in laser-treated ROP cases, others found no significant differences in AL [6,23,24]. Delayed ocular development due to prematurity has been linked to shorter AL, steeper corneal curvature, shallower ACD, and anteriorly positioned lenses, contributing to myopia [14]. Fieß et al. [9] found that decreasing GA was associated with reduced ACV, and BW correlated positively with ACD and CD. Similarly, Wu et al. [13] reported a smaller corneal diameter in preterms compared to term-born controls, with CD increasing with BW. In our study, while AL and ACD differed between laser-treated and other groups, no difference was observed between untreated preterms and term-born controls. Chou et al. [25] also reported shorter AL in laser-treated cases, while Chang et al. [6] found narrower ACD and iridocorneal angles in laser-treated eyes. Farvardin et al. [12] demonstrated smaller ACD and AL in laser-treated cases compared to regressed ROP cases at age 6. Differences across studies may be due to variations in patient demographics, follow-up durations, and measurement techniques.

It is crucial to note that the biometric pattern observed in the laser-treated ROP group is more consistent with myopia of prematurity (MOP) than with classical axial myopia. In these eyes, the refractive error is primarily influenced by altered ocular development and anterior segment configuration, rather than axial elongation alone, as indicated by the combination of shortened axial length, steeper corneal curvature, shallower anterior chamber depth, and higher SRK-T lens power [14].

Our findings may be consistent with an anterior shift in the iris-lens diaphragm and alterations in anterior segment configuration in the laser-treated ROP group. These changes may be associated with reduced ACD and ACV, as well as increased myopia and SRK-T lens power. Nevertheless, the specific lenticular contribution is speculative due to the absence of direct measurement of lens thickness. The anterior shift may, in turn, be related to peripheral displacement of corneal collagen lamellae, contributing to central corneal thinning, steepening, and increased corneal astigmatism and topographic indices. However, mechanistic explanations such as fibrosis and tissue remodeling remain speculative and cannot be confirmed by our data.

Accordingly, it is probable that the more pronounced alterations observed in the laser-treated group are a result of the increased severity of prematurity and treatment-requiring ROP, rather than an independent effect of laser treatment. Despite the fact that a contributory function of laser treatment cannot be entirely disregarded, the current study design does not enable the separation of its independent effect from the influence of disease severity. Wu et al. [13] proposed that prematurity may be associated with alterations in corneal biomechanics, with laser therapy further exacerbating corneal steepness and anterior astigmatism. Overall, these findings are more consistent with developmental variations in anterior segment structure rather than a distinct corneal disease mechanism, and further histopathological and longitudinal studies are needed to clarify the underlying processes.

The strengths of our study include clear group categorization and the use of corneal tomography, optical biometry, and autorefractometry to analyze parameters separately, enhancing result reliability. Unlike previous studies, we examined an older age group, providing insights into the long-term effects of ROP and its treatments.

Our study’s limitations include its single-center design and challenges in obtaining high-quality imaging in some cases due to conditions like cerebral palsy and amblyopia. The absence of lens thickness data and the unavailability of Belin–Ambrosio enhanced ectasia display/index values for all included patients, along with the relatively small number of untreated ROP and laser-treated cases, may have influenced the findings and limited the tomographic interpretation. In addition, both eyes were included when eligible, and a small number of children contributed only one eye because high-quality imaging could not be obtained bilaterally. Although this approach allowed retention of sample size in a relatively limited pediatric cohort, it may have introduced within-subject inter-eye correlation and some imbalance in eye-level representation. Additionally, the untreated preterm group was clinically heterogeneous because it included both children without ROP and children with regressed, untreated ROP. This grouping may have reduced the interpretability of comparisons involving this group, as the observed findings may reflect a mixture of prematurity-related changes and residual effects of mild ROP. It may also have attenuated potential differences between groups. Another limitation is that the control group was recruited from children who presented to outpatient clinics for routine examinations; only those without a history of preterm birth and without any ophthalmic pathology on examination were included, which may not fully represent the general pediatric population. Furthermore, the laser-treated group had lower gestational age and birth weight and represented the most severe end of the prematurity/ROP spectrum. Therefore, the independent effect of laser treatment could not be separated from the effects of prematurity severity and disease severity.

In conclusion, children with a history of laser-treated ROP exhibited steeper and thinner corneas, greater astigmatism, shorter axial length, and shallower anterior chamber parameters compared to untreated preterm and term-born children. These findings suggest persistent differences in anterior segment development associated with severe prematurity and treatment-requiring ROP, rather than evidence of a specific ectatic corneal disorder. Long-term ophthalmologic follow-up may be beneficial for monitoring refractive and structural changes in this population.

## Figures and Tables

**Table 1:** The demographic characteristics of the study groups.

	Group 1 (n = 27)	Group 2 (n = 59)	Group 3 (n = 58)	*p*
**Age**	8.0 ± 1.1	8.9 ± 1.2	8.8 ± 1.1	**<0.001**
**GA**	29.0 ± 2.9	31.3 ± 2.9	38.8 ± 1.1	**<0.001**
**BW**	1367 ± 551	1734 ± 519	3392 ± 466	**<0.001**
**Gender (n, %)**				
**Male**	16 (59)	32 (54)	33 (57)	
**Female**	11 (41)	27 (46)	25 (43)	
**ROP-positive cases (n)**	27	15		
**zone (n, %)**				
**1**	2 (7)	0 (0.0)		
**2**	23 (85)	12 (80)		
**3**	2 (7)	3 (20)		
**stage (n, %)**				
**1**	0 (0)	12 (80)		
**2**	2 (7)	3 (20)		
**3**	25 (93)	0 (0.0)		
**plus (n, %)**	25 (93)	0 (0.0)		

BW, birth weight; GA, gestational age; ROP, retinopathy of prematurity. Bold formatting is used to indicate statistically significant results (*p* < 0.05).

**Table 2:** Comparison of refractive status among study groups.

	Group 1	Group 2	Group 3	*p* *
**Spheric Equivalent (D)**	−0.22 ± 2.82 (−8.25–5.88)	1.04 ± 1.40 (−4.25–4.50)	0.71 ± 1.59 (−4.0–6.13)	0.085
**Myopia** ≤ −**1.00 D, n (%)**	13 (24.1)	13 (11.1)	12 (10.5)	**0.036**
**Myopia** ≤ −**3.00 D, n (%)**	7 (13.0)	2 (1.7)	2 (1.8)	**0.001**
**Hyperopia ≥ +1.00 D, n (%)**	19 (35.2)	63 (53.8)	49 (43)	0.053
**Hyperopia ≥ +3.00 D, n (%)**	2 (3.7)	5 (4.3)	7 (6.1)	0.726
**Astigmatism ≥ 1.00 D, n (%)**	33 (61.1)	19 (16.2)	34 (29.8)	**<0.001**
**Astigmatism ≥ 3.00 D, n (%)**	7 (13.0)	1 (0.9)	4 (3.5)	**0.001**

* Chi-square test, *p* < 0.05. Values for spherical equivalent are presented as mean ± standard deviation with corresponding ranges (minimum–maximum); other variables are given as number (%), unless otherwise specified. n indicates the number of eyes; D, diopter. Bold formatting is used to indicate statistically significant results (*p* < 0.05).

**Table 3:** Corneal tomography parameter comparisons among the study groups.

	Group	Min	Median	Max	Mean ± SD	*p* *	*p* **
**Kh**	1	42.2	43.9	47.8	44.40 ± 1.44	<0.001	**1–2: <0.001**
**(D)**	2	39.2	43.3	45.8	43.25 ± 1.45		**1–3: <0.001**
	3	39.4	42.5	46.5	42.61 ± 1.58		2–3: 0.002
**Kv**	1	42.7	45.5	51.4	46.02 ± 1.79	<0.001	**1–2: <0.001**
**(D)**	2	40.2	44.2	47.0	43.93 ± 1.73		**1–3: <0.001**
	3	40.4	43.6	48.5	43.81 ± 1.67		2–3: 0.067
**Km**	1	42.6	44.6	49.5	45.19 ± 1.57	<0.001	**1–2: <0.001**
**(D)**	2	39.7	43.7	46.4	43.74 ± 1.36		**1–3:<0.001**
	3	39.9	43.1	47.3	43.19 ± 1.60		2–3: 0.012
**Kmax**	1	43.0	46.1	52.2	46.56 ± 1.91	<0.001	**1–2: <0.001**
**(D)**	2	41.2	44.6	47.6	44.77 ± 1.40		**1–3: <0.001**
	3	40.8	43.95	49.6	44.30 ± 1.74		2–3: 0.046
**Ant Ast**	1	0.2	1.6	3.6	1.62 ± 0.85	<0.001	**1–2: <0.001**
**(D)**	2	0.2	0.9	3.4	1.00 ± 0.63		**1–3: 0.007**
	3	0.1	1.1	3.3	1.20 ± 0.66		**2–3: 0.025**
**Post Ast**	1	0.2	0.3	0.8	0.35 ± 0.16	0.010	**1–2: 0.023**
**(D)**	2	0.0	0.3	0.6	0.28 ± 0.13		1–3: 1.000
	3	0.1	0.3	0.7	0.32 ± 0.14		2–3: 0.050
**CCT**	1	491	547	661	545.8 ± 33.7	<0.001 ‡	**1–2: 0.002 ‡‡**
**(μm)**	2	463	568	636	566.0 ± 38.8		**1–3: <0.001 ‡‡**
	3	499	566	638	567.0 ± 32.1		2–3: 0.973 ‡‡
**CVT**	1	491	549	662	546.9 ± 34.3	<0.001 *	1–2: <0.001 **
**(μm)**	2	463	571	637	566.5 ± 38.8		1–3: <0.001 **
	3	501	567	639	567.7 ± 32.1		2–3: 1.000 **
**TCT**	1	490	545	659	543.9 ± 33.3	<0.001 ‡	**1–2: 0.002 ‡‡**
**(μm)**	2	462	565	634	563.9 ± 38.9		**1–3: <0.001 ‡‡**
	3	498	563	638	565.2 ± 32.3		2–3: 0.961 ‡‡
**CV**	1	55.5	62.5	77.1	63.00 ± 3.88	0.081 *	
**(mm)**	2	52.0	63.6	73.4	63.84 ± 4.47		NA
	3	55.3	62.7	70.7	62.90 ± 3.43		
**WTW**	1	10.2	11.5	12.9	11.43 ± 0.49	<0.001 *	1–2: 0.008 **
**(mm)**	2	10.4	11.7	12.9	11.67 ± 0.45		1–3: <0.001 **
	3	10.0	11.9	12.9	11.89 ± 0.50		2–3: 0.003 **
**ACV**	1	64.4	138	215	145.7 ± 33.6	<0.001 *	1–2: <0.001 **
**(mm^3^)**	2	113	177	265	181.9 ± 35.1		1–3: <0.001 **
	3	115	191	265	189.4 ± 33.9		2–3: 0.419 **
**ACD**	1	2.00	2.67	3.39	2.74 ± 0.32	<0.001 ‡	**1–2: <0.001 ‡‡**
**(mm)**	2	2.48	3.07	3.76	3.06 ± 0.28		**1–3: <0.001 ‡‡**
	3	2.42	3.02	3.75	3.04 ± 0.28		2–3: 0.925 ‡‡

* Kruskal–Wallis; ** Kruskal–Wallis post hoc Dunn’s test; ‡ ANOVA F; ‡‡ post hoc ANOVA Bonferroni. ACD, anterior chamber depth; ACV, anterior chamber volume; Ant Ast, anterior astigmatism; CCT, central corneal thickness; CV, corneal volume; CVT, corneal vertex thickness; Kh, horizontal keratometry; Km, mean keratometry; Kmax, maximum keratometry; Kv, vertical keratometry; Post Ast, posterior astigmatism; TCT, thinnest corneal thickness; WTW, white-to-white distance. Bold formatting is used to indicate statistically significant results (*p* < 0.05).

**Table 4:** Comparison of Pentacam Keratoconus Index Values among study groups.

	Group	Min	Median	Max	Mean ± SD	*p* *	*p* **
	1	12	24	45	24.54 ± 6.72		1–2: <0.001 **
**ISV**	2	11	17	34	18.29 ± 4.68	<0.001 *	1–3: <0.001 **
	3	9	17.5	42	18.93 ± 6.16		2–3: 1.000 **
	1	0.05	0.17	0.32	0.17 ± 0.07		1–2: 0.012 **
**IVA**	2	−0.1	0.13	0.29	0.13 ± 0.06	0.008 *	1–3: 0.016 **
	3	0.03	0.13	0.34	0.14 ± 0.06		2–3: 1.000 **
	1	0.98	1.03	1.08	1.03 ± 0.02		1–2: <0.001 **
**KI**	2	0.98	1.02	1.07	1.02 ± 0.02	<0.001 *	1–3: 0.001 **
	3	0.96	1.02	1.07	1.02 ± 0.02		2–3: 0.973 **
	1	1.00	1.01	1.02	1.01 ± 0.01		1–2: 0.001 **
**CKI**	2	0.99	1.01	1.02	1.01 ± 0.01	0.001 *	1–3: 0.276 **
	3	1.00	1.01	1.02	1.01 ± 0.01		2–3: 0.058 **
	1	0.4	5.25	30.5	6.89 ± 5.96		
**IHA**	2	0	4.7	17.6	5.45 ± 3.87	0.399 *	NA
	3	0	4.9	23.3	5.58 ± 4.54		
	1	0.004	0.017	0.041	0.02 ± 0.01		1–2: 0.27 **
**IHD**	2	0.002	0.015	0.037	0.02 ± 0.01	0.024 *	1–3: 0.02 **
	3	0.001	0.0125	0.032	0.01 ± 0.01		2–3: 0.586 **
	1	6.47	7.33	7.85	7.26 ± 0.30		1–2: <0.001 **
**Rmin**	2	7.1	7.56	8.18	7.55 ± 0.23	<0.001 *	1–3: <0.001 **
	3	6.8	7.68	8.27	7.63 ± 0.29		2–3: 0.038 **
	1	0.48	4.62	45.35	7.81 ± 9.95		1–2: 0.135 **
**CSAI**	2	0.33	6.5	53.33	10.20 ± 11.10	0.043 *	1–3: 1.000 **
	3	0.33	5.18	49.03	7.75 ± 8.69		2–3: 0.087 **
	1	−1.17	0.3	1.62	0.3 ± 0.6		**1–2: <0.001 ‡‡**
**IS**	2	−1.81	−0.16	1.47	−0.1 ± 0.6	<0.001 ‡	1–3: 0.087 ‡‡
	3	−1.75	0.04	1.74	0.1 ± 0.6		**2–3: 0.027 ‡‡**

* Kruskal–Wallis; ** Kruskal–Wallis post hoc Dunn’s test; ‡ ANOVA F; ‡‡ Post hoc ANOVA Bonferroni. CKI, central keratoconus index; CSAI, corneal surface asymmetry index; IHA, index of height asymmetry; IHD, index of height decentration; IS, inferior–superior index; ISV, index of surface variance; IVA, index of vertical asymmetry; KI, keratoconus index; Rmin, minimum radius of curvature. Bold formatting is used and to indicate statistically significant results (*p* < 0.05).

**Table 5:** Comparison of IOL Master measurements among the study groups.

	Group	Min	Median	Max	Mean ± SD	*p* *	*p* **
	1	20.3	21.9	25.3	22.04 ± 1.18		1–2: <0.001 **
**AL (mm)**	2	21.4	22.8	25.3	22.89 ± 0.89	<0.001 *	1–3: <0.001 **
	3	20.7	22.9	25.1	22.96 ± 1.01		2–3: 1.000 **
	1	42.7	44.2	48.2	44.77 ± 1.50		1–2: <0.001 **
**Kh (D)**	2	39.4	43.5	46.4	43.60 ± 1.48	<0.001 *	1–3: <0.001 **
	3	39.6	42.8	46.9	42.83 ± 1.62		2–3: 0.001 **
	1	43.4	46.1	51.8	46.46 ± 1.85		1–2: <0.001 **
**Kv (D)**	2	40.9	44.5	47.7	44.65 ± 1.38	<0.001 *	1–3: <0.001 **
	3	40.4	44.0	49.2	44.11 ± 1.72		2–3: 0.017 **
	1	−3.9	−1.6	−0.2	−1.69 ± 0.95		1–2: <0.001 **
**Ast (D)**	2	−3.9	−1.0	−0.1	−1.07 ± 0.68	<0.001 *	1–3: 0.015 **
	3	−3.7	−1.1	−0.1	−1.21 ± 0.86		2–3: 0.113 **
**SRK-T Lens** **Power (D)**	1	14	25	29.5	24.24 ± 3.57		1–2: <0.001 **
	2	16	23	28.0	22.80 ± 2.18	<0.001 *	1–3: 0.011 **
	3	16.5	23.5	29.5	23.29 ± 2.54		2–3: 0.222 **
	1	2.07	3.17	3.99	3.19 ± 0.37		**1–2: <0.001 ‡‡**
**ACD (mm)**	2	2.88	3.56	4.12	3.56 ± 0.26	<0.001 ‡	**1–3: <0.001 ‡‡**
	3	3.01	3.58	4.14	3.54 ± 0.25		2–3: 0.924 ‡‡

*; ** Kruskal–Wallis post hoc Dunn’s test; ‡ ANOVA F; ‡‡ Post hoc ANOVA Bonferroni. ACD, anterior chamber depth; AL, axial length; Ast, astigmatism; Kh, horizontal keratometry; Kv, vertical keratometry; SRK-T Lens Power, Sanders–Retzlaff–Kraff theoretical lens power. Bold formatting is used to indicate statistically significant results (*p* < 0.05).

## Data Availability

The data presented in this study are available on request from the corresponding author (A.T.B.) due to privacy.
